# Effects of irradiated biological dressings on second-degree burn wounds

**DOI:** 10.4314/ahs.v23i2.41

**Published:** 2023-06

**Authors:** Xia Zhang, Yi Zhang, Yangyang Wu, Qinchen Xia, Yaoyao Ji, Wangwang Yao, Jun Qi, Ling Cao

**Affiliations:** Department of Burn and Plastic, Affiliated Hospital of Nantong University, Nantong 226001, China

**Keywords:** Second-degree burn wounds, irradiated biological dressing, wound healing, effect

## Abstract

**Objective:**

To explore the effect of irradiated biological dressing (IBD) on second degree burn wounds.

**Methods:**

Eighty patients with second-degree burns who were treated in our department were selected and randomly divided into IBD group and traditional dressing (TD) group by random number table method. The dressing change, wound healing, comfort and adverse reactions of patients in the two groups were compared and analysed.

**Results:**

The number of dressing changes in the IBD group was significantly less than that in the TD group, and the pain degree of dressing changes was significantly lower than that in the TD group (P<0.05). The dressing comfort of the IBD group was higher than that of the TD group, the secondary trauma score was lower than that of the TD group, the wound scar hyperplasia score was lower than that of the TD group, and the healing time was shorter than that of the TD group (P<0.05). There was no statistically significant difference in adverse reactions between the two groups (P>0.05).

**Conclusion:**

IBD can promote the healing of second-degree burn wounds, improve patient comfort, reduce secondary trauma and wound scarring, and improve patients' quality of life.

## Introduction

As the first line of defense of the human body, the skin is the largest organ of the human body and plays a very important role. After the burn occurs, the skin is necrotic, and it is difficult to completely remove the wound necrosis in time. The necrotic tissue of the burn wound becomes a good culture medium for bacteria, which can easily lead to infection of the body^1^-^3^. On the one hand, bacterial reproduction consumes nutrients on the wound surface, destroys the new epithelium, and makes the wound difficult to heal; on the other hand, severe infection can cause burn sepsis, which threatens the life of patients^4^, ^5^. Wound healing is an extremely complex and orderly repair process, and the interference of external factors can easily affect the healing speed and effect^6^-^9^. Therefore, it is necessary to seal the burn wound early to reduce the chance of bacterial invasion, and to create positive and favourable conditions for the growth of granulation and the coverage of new epithelium on the wound 10-12.

Wound problem is the fundamental problem for burn patients, and wound care of burn patients is the top priority. Different wound treatment methods have different effects on wound healing. In the treatment of burn wounds, on the one hand, it is necessary to speed up the healing and reduce the scar problem after the wound heals; on the other hand, it is necessary to reduce the degree of pain in the process of wound care. Wound dressing is an important material for the care of burn patients. Traditional dressings (TD) have poor biocompatibility and require frequent dressing changes, which not only affects wound healing, but also brings pain to patients. Irradiated biological dressings (IBD) have the characteristics of low antigenicity and good compatibility with wound tissue^13^. However, its efficacy in the treatment of second-degree burns is rarely reported. In this study, 80 patients with second-degree burns who were treated in our department from March 2020 to March 2022 were selected and randomly divided into IBD group and TD group by random number table method, with 40 patients in each group. This study aimed to explore effect of IBD on second degree burn wounds and provide a strong basis for dressing selection for second-degree burn wounds.

## Methods

This study was approved by the Ethics Committee of the Affiliated Hospital of Nantong University. All patients or guardians signed the informed consent.

### Patients

In this study, 80 patients with second-degree burns who were treated in our department from March 2020 to March 2022 were selected and randomly divided into IBD group and TD group by random number table method, with 40 patients in each group.

**Inclusion criteria:** (1) The depth of burns is second-degree, and the burn area is 3% to 30%; (2)The patients volunteered to participate in this researcher, and the clinical data were complete; (3)No mental illness or disorder of consciousness.

**Exclusion criteria:** (1) Patients with skin disease before burn; (2) Wounds with exposed bone, joint capsule and tendon; (3) Progressive wound depth; (4) Wounds located on the face and perineum; (5) Combined with diabetes and immune system diseases, metabolic disorders, etc.; (6) Combined with serious diseases of the heart, liver, kidney and other important organs; (7) Those who were allergic to drugs.

### Treatment

Both groups of patients received intravenous infusion of antibiotics to prevent infection (empirical antibiotics were used in the early stage, and sensitive antibiotics could be selected according to the wound surface and blood culture results in the later stage), and internal environmental factors such as acid-base imbalance and electrolyte imbalance were corrected at the same time. If there were other complications, such as inhalation injury, treated symptomatically. Except for different wound dressings, the other nursing methods for patients in the two groups were the same. Wound cultures were performed weekly to monitor infection.

**Wound care of TD group:** First wash the normal skin around the wound with soap and warm water, then shave the hair around the wound. The wounds were disinfected with 0.5% povidone-iodine, washed with normal saline, and dried with sterile gauze. For patients with intact blisters on superficial second-degree wounds, they should be cut at the low position to remove the blisters and retain the blisters. For patients with incomplete blisters on superficial grade II wounds and deep grade II wounds, putrefied skin was removed. According to the depth and contamination of the wound surface, different functional dressings were selected, covered with Vaseline gauze, and fixed with sterile gauze and bandage, and the dressing range exceeded the wound edge by 5 cm. The bandaging of the limbs generally started from the distal end of the limb, and the functional position was bandaged. During the bandaging process, attention should be paid to the appropriate tightness, and the distal segments of the fingers (toes) were exposed to facilitate the observation of peripheral circulation. During the trunk bandaging process, paid attention to listening to the patient's chief complaint and observed the patient's breathing, especially the patient with inhalation injury. After debridement, the affected limb should be elevated by 15-30 degrees, and the body should be turned over every 2-3 hours to avoid prolonged pressure on the wound surface, kept the wound dressing clean and dry, and prevented infection. Dressing should be changed according to the exudation of the wound. During the nursing process, hand hygiene, aseptic technique principles, and disinfection and isolation specifications should be strictly implemented.

**Wound care of IBD group:** The method of wound debridement was the same as that of the TD group. After debridement, the IBD (Tianjin TOEFL Medical Atomic Energy Technology Co., Ltd.) was used to cover the wound. IBD were generally stored in a freezer at -18^0^ and have a validity period of 24 months. Before use, the IBD was rewarmed and softened at 35^0^, then applied the folded inner surface directly to the wound, pasted it flat, leaved no air bubbles, and used sterile gauze and bandage for external dressing. After three days, the dressing was changed for the first time, and the adhesion between the IBD and the wound surface was observed. If the adhesion was satisfactory, bandaged again until it healed; if there was more local exudation, a small incision should be cut in time for drainage. The IBD has been perforated during the production process to facilitate drainage of exudate. The time and frequency of changing the external dressing was determined according to the exudation until the IBD fallen off and the wound healed. Bandaging and nursing precautions were the same as those in the TD group.

### Observation Indicator

(1) Number of dressing changes; (2) Comfort during dressing changes: The degree of pain of the patient during dressing change was used to evaluate the comfort. The patients were instructed to use the numerical rating scale (NRS) evaluation, with a score ranging from 0 to 10, with 0 being no pain and 10 being unbearable severe pain (requiring analgesic drug intervention). Among them, 1-3 was mild pain(I), 4-6 was moderate pain (II), and ≥7 was severe pain (III). (3) Comfort of dressing coverage: It was evaluated with the same method and standard of comfort during dressing change. (4) Secondary wounds score: It was evaluated according to the World Union of Wound Healing Societies (WUWHS) evaluation standard for dressings^14^. 1 point: the dressing does not adhere to the wound surface, and no secondary wound and bleeding occur; 2 points: the dressing is partially adhered to the wound surface, and there are secondary wounds, but small in scope, with a small amount of bleeding; 3 points: The dressing is seriously adhered to the wound surface and needs to be removed and soaked. The secondary wound is flaky and requires compression to stop bleeding. (5) Wound healing time: The wound healing standard was the presence of new epithelial tissue coverage and no exudation; (6) Wound scar hyperplasia: All patients were followed up for 6 months, and the wound scar hyperplasia was evaluated according to the Vancouver Scar Scale (VSS)^15^. The scale includes four aspects: blood vessel distribution (0-3 points), color (0-3 points), thickness (0-4 points), and softness (0-5 points). The scoring range is 0-15 points, and the higher the value, the more obvious the scar. (7) Adverse reactions: including wound tingling, wound redness and swelling, and rash.

### Statistical processing

SPSS 21.0 was used to analyse the data obtained in this study. The measurement data in this study conformed to a normal distribution and were expressed as mean±SD, and the independent samples t test was used for comparison between groups. The count data was expressed as percentage, and the chi-square test was used for comparison between groups. P<0.05 considered the difference to be statistically significant.

## Results

### Comparison of dressing changes between groups

**Figure 1:**
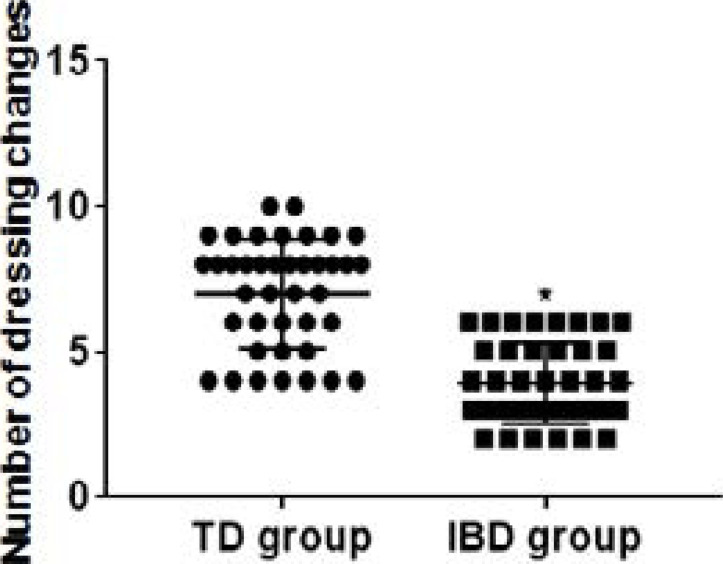
Comparative statistical chart of dressing changes number between groups. * vs.TD group, P<0.05

The number of dressing changes in the IBD group was significantly less than that in the TD group (Fig.1), and the pain degree of dressing changes was significantly lower than that in the TD group, and the difference between the groups was statistically significant (P<0.05) (Table-1).

**Table 1:** Comparison of dressing comfort between two groups (n, %)

	n	I	II	m
TD group	40	18	13	9
IBD group	40	35	4	1
*χ^2^* value		16.16	4.78	3.89
*P* value		< 0.01	0.03	0.05

### Comparison of wound healing and dressing comfort between groups

**Figure 2:**
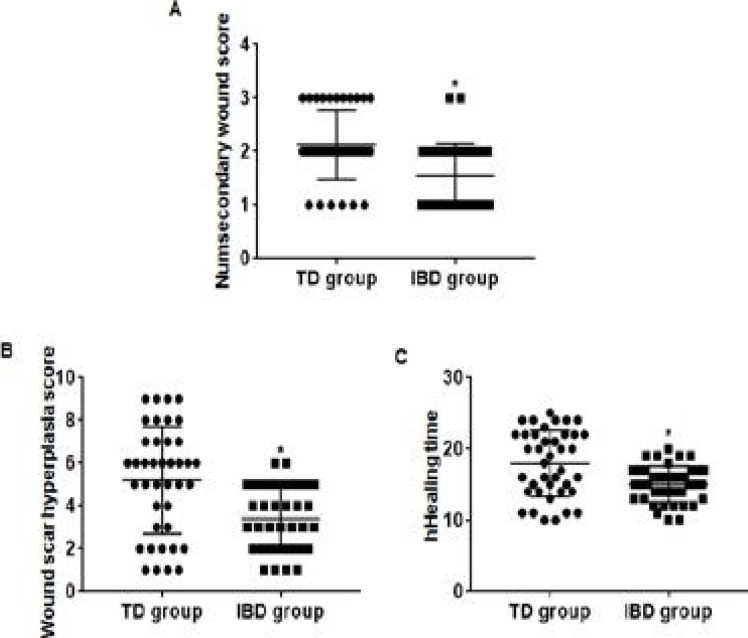
Comparative statistical chart of wound healing between groups. (A) The secondary wound score of the IBD group was significantly lower than that of the TD group. (B) The wound scar hyperplasia score was significantly lower than that of the TD group. (C) The healing time was significantly shorter than that of the TD group. * vs.TD group, P<0.05

The secondary wound score of the IBD group was significantly lower than that of the TD group (Fig.2A), the wound scar hyperplasia score was significantly lower than that of the TD group (Fig.2B), the healing time was significantly shorter than that of the TD group (Fig.2C), and the comfort of dressing coverage was significantly higher than that of the TD group (Table-2), and the differences between the groups were statistically significant (P<0.05).

**Table 2:** Comparison of dressing coverage comfort between two groups (n, %)

	n	I	II	m
TD group	40	6	21	13
IBD group	40	24	12	4
*χ^2^* value		17.28	4.18	4.78
*P* value		< 0.01	0.04	0.03

### Comparison of adverse reactions between groups

The adverse reactions in the two groups mainly included wound tingling, wound redness and swelling, and rash. The difference between groups was not statistically significant (Table-3).

**Table 3:** Comparison of adverse reactions between two groups (n, %)

	n	wound tingling	wound redness and swelling	rash
TD group	40	1	2	1
IBD group	40	2	1	1
*χ^2^* value		0.00	0.00	0.00
*P* value		1.00	1.00	1.00

## Discussion

Burns are tissue damage caused by heat, which is a common accidental skin injury in life. How to promote wound healing and reduce scar hyperplasia in burn patients has always been a key problem to be overcome. After active and effective treatment of second-degree burn wounds, wounds can heal themselves relying on their own regeneration ability. When treating patients with wounds, it is necessary not only to close the wounds as soon as possible to promote the recovery of their tissue structure and function, but also to fully consider the intrusion of pain on the wounds and the psychological impact of the patients. In the conventional treatment plan, it cannot effectively seal the wound surface and reduce the extravasation of body fluids on the wound surface after burn, and this method has many dressing changes, which is easy to stimulate the wound surface and damage the new epithelium, resulting in obvious scar after wound healing, which affects the appearance. At the same time, when changing dressings, gauze can stimulate the subcutaneous tissue where pain sensation is densely distributed, which can bring pain to patients, and even lead to psychological disorders and mental symptoms in patients. According to research^16^-^18^, an ideal wound dressing should have the following conditions: (1) Keep the wound moist and protect the wound; (2) Prevent bacterial invasion; (3) Good adhesion and less pain in dressing change; (4) Good biocompatibility, no toxicity and no antigenicity; (4) Promote wound repair without causing inflammatory reaction.

In order to promote the good and fast healing of second-degree burn wounds, our department has carried out related research using IBD. Our findings are basically consistent with those of other scholars^16^, ^19^-^23^. Compared with conventional treatment, IBD can not only reduce the number of dressing changes, reduce the pain degree during dressing changes, and have better comfort, but also reduce the degree of secondary wound and wound scarring, and shorten the wound healing time. This suggests that the IBD is an ideal wound dressing with remarkable effect. IBD are obtained by processing fresh pig skins into thin skin sheets, soaking them with compound silver-containing medicines, and then irradiating and sterilizing with cobalt 60 and other high-tech bioengineering technologies. Pigs are mammals. Porcine skin has a high homology with humans. It is similar to human skin in structure and collagen content. After irradiation, cells that are prone to immune rejection are removed. It has good biocompatibility with the human body and can reduce rejection, and has the therapeutic functions of analgesia, anti-infection and promoting the growth of wound epithelium^24^. The IBD has good air permeability, less effusion formation, and is not easy to adhere to the wound surface, and thus less secondary wound during dressing change, which can provide a good environment for the growth of granulation on the wound surface, and can also reduce the stimulation to the new epithelium, reducing the occurrence of wound scarring. However, it is worth noting that biological dressings have unique advantages for second-degree burn wounds, but because they have no active bactericidal effect, they may be not suitable for wounds complicated by infection.

In summary, IBD can promote the healing of second-degree burn wounds, improve patient comfort, reduce secondary trauma and wound scarring, and improve patients' quality of life.

## Declarations

### Availability of data and materials

The datasets used and/or analysed during the current study are available from the corresponding author on reasonable request.
